# Perspectives in immunotherapy: meeting report from the “Immunotherapy Bridge” (December 4th–5th, 2019, Naples, Italy)

**DOI:** 10.1186/s12967-020-02627-y

**Published:** 2021-01-06

**Authors:** Paolo A. Ascierto, Lisa H. Butterfield, Katie Campbell, Bruno Daniele, Michael Dougan, Leisha A. Emens, Silvia Formenti, Filip Janku, Samir N. Khleif, Tomas Kirchhoff, Alessandro Morabito, Yana Najjar, Paul Nathan, Kunle Odunsi, Akash Patnaik, Chrystal M. Paulos, Bradley I. Reinfeld, Heath D. Skinner, John Timmerman, Igor Puzanov

**Affiliations:** 1 Cancer Unit of Melanoma, Cancer Immunotherapy and Development Therapeutics, Cancer Immunotherapy and Innovative Therapy, Istituto Nazionale Tumori IRCCS “Fondazione G. Pascale”, Via Mariano Semmola, 80131 Naples, Italy; 2 PICI Research & Development, Parker Institute for Cancer Immunotherapy, San Francisco, CA USA; 3 Oncology Unit, Ospedale del Mare, Naples, Italy; 4 Massachusetts General Hospital, Harvard Medical School, Boston, MA USA; 5 UPMC Hillman Cancer Center, University of Pittsburgh, Pittsburgh, PA USA; 6 Sandra and Edward Meyer Cancer Center, Department of Radiation Oncology, Weill Cornell Medicine, New York, NY USA; 7 Division of Cancer Medicine, Department of Investigational Cancer Therapeutics, The University of Texas MD Anderson Cancer Center, Houston, TX USA; 8 The Loop Immuno-Oncology Research Laboratory, Lombardi Cancer Center, Georgetown University, Washington, DC, USA; 9 Perlmutter Cancer Center, New York University School of Medicine, New York, NY USA; 10 Thoracic Medical Oncology, National Cancer Institute, IRCCS-Fondazione G. Pascale, Naples, Italy; 11 Mount Vernon Cancer Centre, Northwood, UK; 12 Center for Immunotherapy and Department of Gynecologic Oncology, Roswell Park Comprehensive Cancer Center, Buffalo, NY USA; 13 Section of Hematology and Oncology, Department of Medicine, University of Chicago, Chicago, IL USA; 14 Winship Cancer Institute at Emory University, Atlanta, GA USA; 15 Vanderbilt University Medical Center, Nashville, TN USA; 16 Department of Radiation Oncology, UPMC Hillman Cancer Center, University of Pittsburgh, Pittsburgh, PA USA; 17 University of California, Los Angeles, Los Angeles, CA USA; 18 Roswell Park Comprehensive Cancer Center, Buffalo, NY USA

**Keywords:** Immunotherapy, Checkpoint inhibitors, Combination therapy, Biomarkers, Tumor microenvironment, Vaccine

## Abstract

Over the last few years, numerous clinical trials and real-world experience have provided a large amount of evidence demonstrating the potential for long-term survival with immunotherapy agents across various malignancies, beginning with melanoma and extending to other tumours. The clinical success of immune checkpoint blockade has encouraged increasing development of other immunotherapies. It has been estimated that there are over 3000 immuno-oncology trials ongoing, targeting hundreds of disease and immune pathways. Evolving topics on cancer immunotherapy, including the state of the art of immunotherapy across various malignancies, were the focus of discussions at the Immunotherapy Bridge meeting (4–5 December, 2019, Naples, Italy), and are summarised in this report.

## Introduction

Over the last few years, extensive research has improved our understanding of tumour immunology and enabled the development of novel treatments that can harness the patient’s immune system and prevent immune escape. Through numerous clinical trials and real-world experience, a large amount of evidence demonstrating the potential for long-term survival with immunotherapy agents has been accumulated across various malignancies, beginning with melanoma and extending to other tumours.

The results of these studies have also highlighted a number of recurring observations with immuno-oncology agents, including their potential for clinical application across a broad patient population and for both conventional and unconventional response patterns. The clinical success of immune checkpoint blockade with anti-cytotoxic T-lymphocyte-associated antigen (CTLA)-4 and anti-programmed death (PD)-1/PD-ligand (L)1 inhibitor has encouraged increasing development of other immunotherapies, particularly monoclonal antibodies with other immune targets, adoptive cell transfer and vaccines. It has been estimated that there are over 3000 immuno-oncology trials ongoing, targeting hundreds of disease and immune pathways.

Evolving topics on cancer immunotherapy, including the state of the art of immunotherapy across various malignancies, were the focus of discussions at the Immunotherapy Bridge meeting (4–5 December, 2019, Naples, Italy), and are summarised in this report.

## SITC session

## Mechanisms of success and failure in immunotherapy

### Successes and failures in cell therapies for solid tumors

There have clearly been many successes with T cell therapies in recent years. However, while CD19-targeted and other chimeric antigen receptor (CAR) T cell therapies have elicited long-lasting and even curative responses in many patients, to date their application has not been effective in treating solid tumors. Given that these constitute the vast majority of cancers, this represents a major unmet need.

In addition, while checkpoint blockade therapy can improve survival of patients with several types of solid tumors, many patients either do not respond or relapse. One approach to improve outcomes for these checkpoint inhibitor-resistant patients may be the use of adoptive cell transfer (ACT) to develop T cell therapies. Tumor-infiltrating lymphocyte (TIL)-based approaches may be more useful than CAR T therapies given the heterogeneity of solid tumors and presence of multiple antigens. However, TIL expansion requires considerable time. This is less of an issue in manufacturing CAR T cells, for which the expansion process is quicker, although expensive. One solution may be to use more potent T cells that can be rapidly expanded.

CD4+ helper T cells play a key role in immunity. CD26 expression levels can be used to identify three human CD4 subsets (CD26^neg^, CD26^int^ and CD26^high^) [1]. CD26^neg^ T cells have a regulatory phenotype while CD26^int^ T cells are mainly naïve. However, CD26^high^ T cells have stem-cell like properties and can persist and proliferate in solid tumors. CD26^high^ cells also have a rich chemokine receptor profile (including CCR2 and CCR5), profound cytotoxicity (Granzyme B and CD107A), resistance to apoptosis (c-KIT and Bcl2), and enhanced stemness (β-catenin and Lef1). CD26^high^ T cells also co-secrete multiple effector cytokine, including interleukin (IL)-2, interferon (IFN)-γ, IL-17A, IL-22, and tumor necrosis factor (TNF)-α. These properties mean CD26^high^ T cells have the ability to traffic to, regress and survive in solid tumors. The higher the CD26 expression, the more therapeutic the CD4+ cells. T cells that express high levels of CD26 have been shown to effectively regress tumors in a human mesothelioma mouse model. Similarly, in a mouse pancreatic cancer model CD26^high^ T cells significantly slowed the progression of tumors after CAR transduction, whereas bulk CD4+ and CD26^neg^ T cells yielded little-to-no antitumor response. Decreased tumor growth and weight was also observed in mice treated with CD26^high^ T cells compared to mice treated with CD4+ or CD26^neg^ T cells. A CD26+ CAR-T specific for mesothelin, which is highly expressed in several human cancers including malignant mesothelioma, pancreatic cancer and lung adenocarcinoma, is currently being developed. This offers the potential benefits of significantly improved persistence, high migratory capacity to tumors and polyfunctional cytokine expression, leading to improved potency.

### Leveraging multiomic data to accelerate translational impact at the Parker Institute for Cancer Immunotherapy

Challenges in next generation sequencing from clinical samples include optimizing nucleic acid extraction and sequencing strategies and then bridging these with various -omics analysis workflow pipelines.

Formalin-fixed paraffin-embedded (FFPE) preservation is often used because of its economic feasibility but can compromise both the quality and quantity of nucleic acid extracted from samples. In an experimental comparison to evaluate nucleic acid extraction and various RNA sequencing library preparations from FFPE samples, the Covaris truXTRAC^®^ FFPE tNA Plus kit showed superior RNA yields and quality from less input tissue compared to the Qiagen miRNeasy^®^ FFPE kit and enables concurrent dual DNA and RNA extraction from the same lesion. Optimizing library preparation for RNA sequencing was achieved with the Agilent SureSelect^XT HS^ technology that promoted amplification and capture of longer RNA fragments. All libraries displayed equivalent high alignment rates to the reference genome, varying from 94.54 to 97.91% (mean 96.90%), despite different extraction and library preparation approaches. Gene expression was highly concordant across the various library preparation and extraction techniques, with correlation ranging from 0.98–0.99.

Standardizing integrated-omics analysis across clinical datasets has the objectives of leveraging multiple sequencing and analysis strategies in performing immunogenomics analysis, facilitating congruent analysis efforts and making workflows available across different laboratories. The use of the Terra and Google Cloud Platforms can help achieve standardization.

Automated pipelines for integrated data analysis include RNA sequencing, that may involve gene expression as well as analysis of gene signatures, immune cell deconvolution, gene network/pathway analysis and T cell receptor (TCR) clonotyping. DNA sequencing can include gene methylation status, gene network/pathway analysis based on methylation data and correlation with gene expression. Somatic variant detection and germline variant detection may also be included. Somatic and germline analysis in particular have considerable well annotated information on the clinical implications of certain variants with many databases available. Reporting of known clinically actionable variants can be automated and this information needs to be adequately communicated to physicians. Various patient-specific information and cohort metadata, e.g. differential expression analysis, clonal dynamics, can also be inputted.

In conclusion, we need to emphasise the importance of sample collection, processing and experimental strategies of studying samples need to be standardised across institutions.

### Successes and failures in checkpoint blockade and costimulation

Targeting PD-1 and CTLA-4 immune checkpoints has resulted in durable responses in cancers that were considered terminal even a decade ago. These treatments have to date been approved in multiple indications, across different lines of therapy. This includes the first biomarker based tumor agnostic approval (MSI-H), and this number is likely to increase still further. However, while checkpoint inhibitors have changed the treatment paradigm for metastatic melanoma and several other cancers, responses only occur in a subset of patients and many patients receive little or no benefit from these treatments.

The tumor microenvironment (TME) has an immunosuppressive landscape and metabolic deregulation of tumor cells may represent a key resistance mechanism to immunotherapy through the formation of a metabolically suppressive microenvironment. High tumor cell oxidative metabolism may be a barrier to PD-1 blockade immunotherapy in melanoma, and high tumor cell oxidative metabolism has been linked decreased progression free and overall survival in melanoma patients treated with anti-PD-1 immunotherapy [2]. Metabolic derangement varies widely between tumor samples (patient-derived cell lines and ex vivo analysis of patient samples), with some tumor cells demonstrating deregulated oxidative or glycolytic metabolism, or both. Elevated tumor oxidative metabolism is associated with increased T cell exhaustion and decreased immune activity. Furthermore, oxidative but not glycolytic metabolism is associated with resistance to anti-PD-1 blockade. Interestingly, enrichment of oxidative phosphorylation has been demonstrated in melanoma brain metastases [3]. These data suggest that the ability of a tumor cell to sequester oxygen rather than its ability to consume glucose from infiltrating T cells is what renders it resistant to anti-PD-1 therapy [2], and may function as a mechanism of tumor escape from immunotherapy. Tumor cell oxidative metabolism may be a target to improve immunotherapeutic response, and clinical trials evaluating remodelling of the tumor microenvironment are ongoing.

**Fig. 1:**
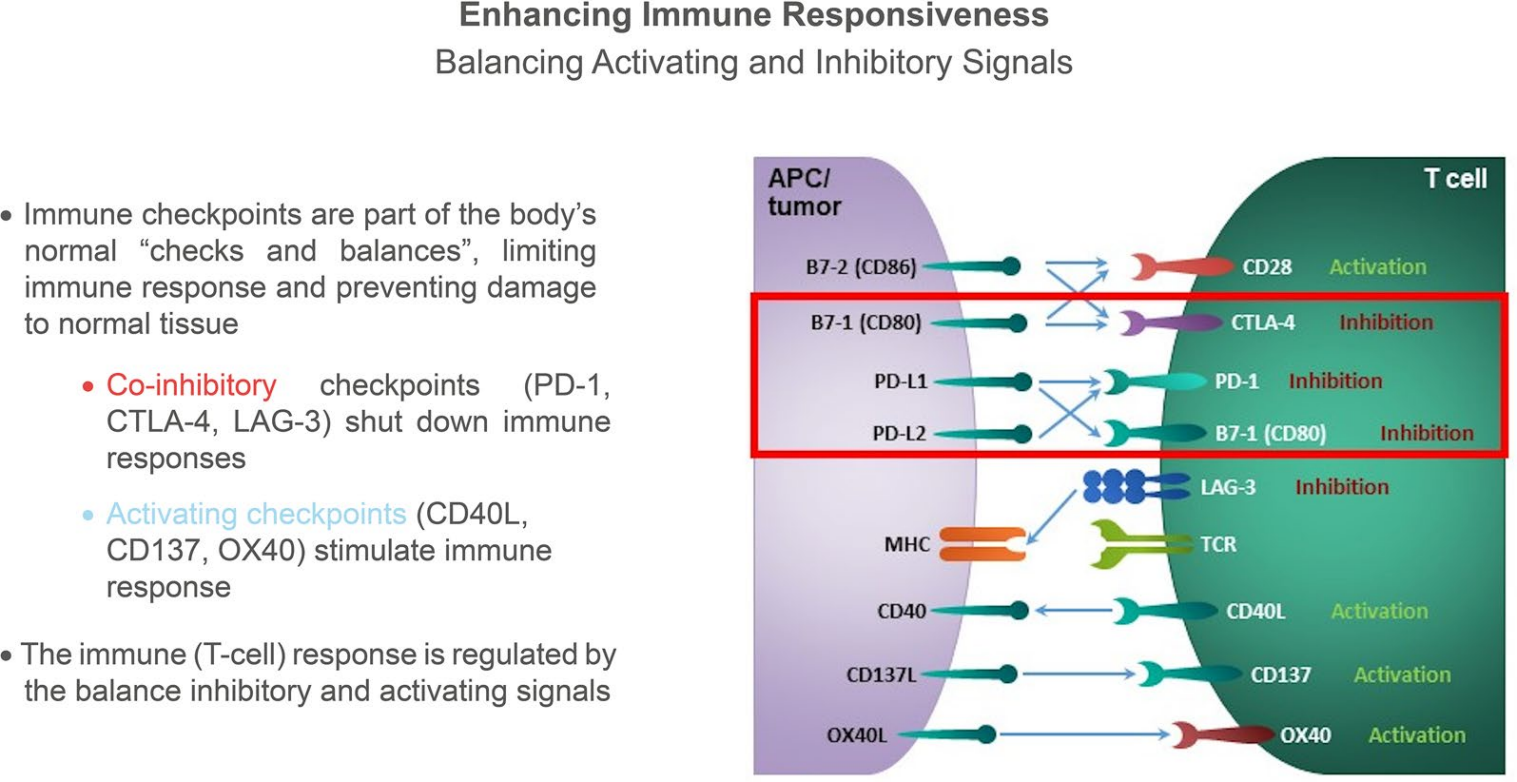
Enhancing immune responsiveness: balancing activating and inhibitory signals

Approaches to extending the benefits of immunotherapy to more patients include third-generation inhibitory targets (e.g. indoleamine 2,3-dioxygenase [IDO], anti-lymphocyte-associated gene [LAG]-3, anti-T-cell immunoglobulin and mucin domain [TIM]-3, anti-T cell immunoreceptor with Ig and ITIM domains [TIGIT]) and costimulatory modalities (e.g. anti-glucocorticoid-induced TNF receptor-related [GITR] protein, anti-CD137 [41BB], anti-OX40, toll-like receptor [TLR]-9 agonist, and anti-CD40L) (Fig. 1).

Multiple studies evaluating the efficacy of third generation inhibitory checkpoints and co-stimulatory pathways are underway, targeting non-redundant pathways. LAG-3 is a cell surface molecule expressed on activated T, B, and NK cells as well as peripheral dendritic cells (DCs) that is increased in exhausted CD8+ T cells. LAG-3 blockade enhances T cell function and preclinical models suggest synergy with anti-PD-1 therapy. In preliminary data, overall response rate (ORR) was 11.5% in patients treated with the anti-LAG-3 agent relatlimab in combination with nivolumab for advanced melanoma that progressed on prior anti-PD-1/PD-L1 therapy [4]. Higher responses correlated with LAG-3 expression, irrespective of PD-L1 expression. TIGIT is present on T and NK cells and regulates T cell mediated immunity. Blockade of TIGIT increases T cell proliferation and cytokine production. TIM-3 is a co-inhibitory receptor expressed on T cells, T regulatory cells (Tregs), and DCs that inhibits Th1 responses and cytokine expression (TNF, IFN-γ). Anti-TIM-3 blockade enhances anti-tumor immunity and suppresses tumor growth.

GITR is part of the NF-R superfamily and has been shown to have increased expression upon T cell activation, and it is thought to play a key role in dominant immunological self-tolerance maintained by CD25+ /CD4+ Tregs. In patients with advanced solid tumors, no responses were seen with the GITR agonist BMS-986156 alone, while in combination with nivolumab, ORRs ranged from 0 to 11.1% (1 of 9) across cohorts, with responses seen in patients previously treated with anti-PD-1 therapy [5].

The development of rational combinations on the basis of pre-clinical and translational data is critical. Timing and sequencing are likely to be key.

### Successes and failures in cancer vaccination

**Fig. 2:**
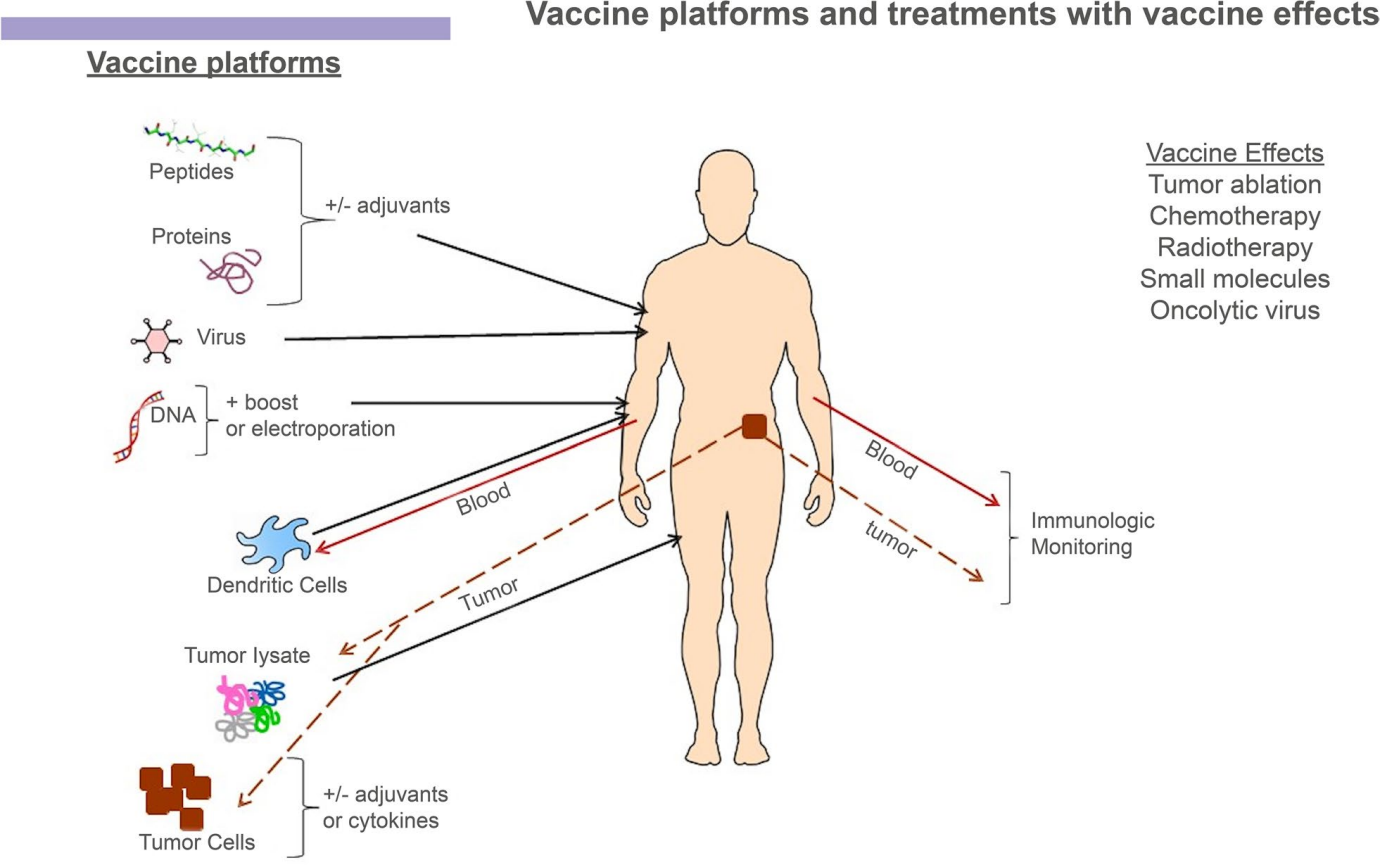
Vaccine platforms and treatments with vaccine effects

Several platforms for cancer vaccination are being tested, including peptides, proteins, tumor cells, DCs and viruses (Fig. 2). Standard of care treatments, such as tumor ablation, chemotherapy, and radiotherapy, can also induce antitumor immunity, thereby having cancer vaccine effects.

DC-based vaccines can promote antitumor immunity and have been shown to result in durable objective responses and improved survival. In an early trial, adenovirus (AdV)-MART-1-engineered DC vaccine resulted in 3 of 14 melanoma patients showing evidence of determinant spreading and longer overall survival (OS) [6]. In a subsequent trial, DCs were engineered with three tumor antigens (tyrosinase, MART-1 and MAGE-A6) to activate multiple CD8 + and CD4+ T cell clones and patients were randomized to receive a one-month course of high-dose systemic IFN-α2b following vaccination or vaccination alone [7]. Of 35 evaluable patients, there were two partial responses (PRs) and eight with stable disease (SD) in the 24 patients with measurable disease while in 11 patients with no evidence of disease at baseline, seven recurred. Most patients showed an increase in vaccine antigen-specific CD8+ and CD4+ T cell responses. The addition of IFN-α did not appear to improve immune or clinical responses. DC vaccine potency testing and immune biomarker profiling identified several significant baseline and on-treatment cellular subsets and circulating soluble proteins that impact both immunologic and clinical outcomes e.g. circulating Tregs and myeloid-derived suppressor cells (MDSCs) were important for the development of antigen-specific T cell responses. responses [8]. Standard DC phenotypic markers do not correlate with clinical outcome. For example, IL-12p70 and IL-10 protein expression levels were not correlated with immune or clinical response [9]. More research is needed to better understand the biology of an effective DC vaccine, the generation of a personal, multi-peptide neoantigen vaccine is another approach being investigated. A vaccine that targets up to 20 predicted personal tumour neoantigens was shown to be feasible in patients with high-risk melanoma [10]. The computational identification of neoantigens is a multistep process and neoepitope pipelines are becoming more common, diverse and complex. The Tumor neoEpitope SeLection Alliance (TESLA) [Wells, D.K. et al., Cell 2020, in press.] brought together key players in the field of neoantigen discovery to elucidate current differences in prediction methodologies, generate high quality epitope validation sets, identify the best algorithm features that predict which tumor neoantigens are recognized by T cells and stimulate an immune response. The results identified a new model which includes MHC binding affinity (< 34 nM), tumor abundance (> 33TPM) and pMHC binding stability (> 1.4H) as critical.

## Session: trends in immunotherapy

### Immunotherapy in lung cancer

The therapeutic approach for the second-line treatment of patients with advanced non-small cell lung cancer (NSCLC) without actionable mutations has been revolutionized by the approval of PD-1/PD-L1 inhibitors. Several clinical trials of checkpoint inhibitors in pretreated NSCLC have shown superior OS versus chemotherapy, with a positive correlation between the expression of PD-L1 and the efficacy of immunotherapy being observed. These benefits are sustained long-term, with pooled data on two clinical trials of nivolumab showing a more than fivefold increase in 5-year OS rate compared to treatment with docetaxel (13.4 vs 2.6%) [11]. Both clinical factors associated with higher probability of response to nintedanib plus docetaxel (e.g. early progression or resistance to first-line therapy, high disease burden) or lower probability of response to nivolumab (e.g. ≥ 5 sites with lesions, bone or hepatic metastases, non-smoker status) and molecular criteria (PD-L1 expression) should be considered in deciding the optimal therapeutic approach for patients with pretreated NSCLC without actionable mutations. In patients with squamous histology, there are additional treatment options with afatinib or ramucirumab plus docetaxel but no validated biomarkers that predict response to these agents. A subset of patients also appears to experience a tumor flare under checkpoint inhibitors, which has been recognized as a novel aggressive pattern of disease termed hyper-progression [12]. This is seen in approximately 10% of patients and seems to be associated with specific genomic alterations, e.g. MDM2 family amplification or EGFR aberrations.

Pembrolizumab is also approved as first-line treatment in NSCLC. In the KEYNOTE-024 study of previously untreated advanced NSCLC patients with PD-L1 expression ≥ 50%, median PFS was 10.3 months with pembrolizumab versus 6.0 months with chemotherapy (HR for disease progression or death, 0.50; p < 0.001) [13]. OS rate at 6 months was significantly improved with pembrolizumab (80.2 versus 72.4%; p = 0.005). First-line pembrolizumab monotherapy continued to demonstrate an OS benefit over chemotherapy after long-term median follow-up of 25 months, despite crossover from the control arm to pembrolizumab as subsequent therapy [14]. In the KEYNOTE-042 trial, pembrolizumab improved OS versus placebo in patients with previously untreated locally advanced or metastatic NSCLC and a PD-L1 tumour proportion score (TPS) of ≥ 1%, and approval was extended to this population [15]. In an interim analysis of the IMpower110 study, atezolizumab showed a significant OS improvement versus chemotherapy in patients with high PD-L1 expression (tumor cell ≥ 50% or immune-infiltrating cell ≥ 10%) [16]. A benefit with atezolizumab was also observed among patients with lower PD-L1 expression, but the criteria for statistical significance were not met at this stage.

Checkpoint inhibitors are also being investigated in combination with chemotherapy in various trials (Table 1). In the KEYNOTE-189 study, addition of pembrolizumab to standard chemotherapy of pemetrexed and a platinum-based drug in patients with previously untreated metastatic non-squamous NSCLC without actionable mutations resulted in significantly longer OS and PFS versus chemotherapy alone [17]. Similarly, addition of pembrolizumab to chemotherapy with carboplatin plus paclitaxel or nab-paclitaxel as first-line treatment in patients with previously untreated squamous NSCLC also resulted in significantly improved OS and PFS [18]. Atezolizumab plus chemotherapy also resulted in a significant survival benefit versus chemotherapy of carboplatin and nab-paclitaxel (median OS 18.6 versus 13.9 months, HR 0.79; p = 0.033) as first-line treatment of patients with stage IV non-squamous NSCLC [19]. It has been suggested that anti-PD-1/PD-L1 therapy combined with chemotherapy may be a better option than PD-1/PD-L1 monotherapy in patients with rapidly progressive disease.

**Table 1.** Checkpoint inhibitors + chemotherapy: results

Study	Author	Treatment	Pts	RR (%)	PD (%)	PFS (months)	OS (months)
KN-189	Gandhi [17]	DDP + Pem + Pembro vs DDP + Pem	616	47.6 vs 18.9, p < 0.001	8.8 vs 17.5	8.8 vs 4.9, HR: 0.52, p = < 0.001	nr vs 11.3, HR: 0.49, p < 0.001
IMp-150	Socinski [91]	Atezo + Beva + CP vs Beva + CP	1202	63.5 vs 48	5.1 vs 8.2	8.3 vs 6.8, HR: 0.62, p < 0.001	19.2 vs 14.7, HR: 0.78, p = 0.02*
IMp-130	West [19]	Atezo + CnP vs CnP	724	49.2 vs 31.9	11 vs 18.1	7 vs 5.5, HR: 0.64, p < 0.0001	18.6 vs 13.9, HR: 0.79, p = 0.033
IMp-132	Papadimitrakopoulou [92]	DDP + Pem + Atezo vs DDP + Pem	578	47 vs 32	–	7.6 vs 5.2, HR: 0.60, p = < 0.0001	18.1 vs 13.6, HR: 0.81, p = 0.0797*
KN-407	Paz-Ares [18]	CP or CnP + Pembro vs CP or CnP	559	58.4 vs 35, p = 0.0004	6.9 vs 15.5	6.4 vs 4.8, HR: 0.56, p < 0.0001	15.9 vs 11.3, HR: 0.64, p = 0.0008
IMp-131	Jotte [93]	Atezo + CP vs Atezo + CnP vs CnP	1021	(B) 49 vs (C) 41	–	6.3 vs 5.6, HR: 0.71, p = 0.0001	14 vs 13.9, HR: 0.96*, p = 0.6

Clinical trials of checkpoint inhibitors with chemotherapy in non-small-cell lung cancer

Another strategy is combining checkpoint inhibitors. In the CheckMate-227 study, median OS was 17.1 months with nivolumab plus ipilimumab and 14.9 months with chemotherapy (p = 0.007) in patients with advanced NSCLC and PD-L1 expression ≥ 1%; 2-year OS rates were 40 and 33%, respectively [20]. An OS benefit was also observed in patients with a PD-L1 expression < 1%. Anti-PD-1/PD-L1 therapy has also been shown to have a role in locally advanced NSCLC after chemo-radiotherapy, as well as in combination with chemotherapy in patients with small-cell lung cancer (SCLC). Ongoing trials are also assessing its role as both adjuvant and neoadjuvant therapy.

### Using neoadjuvant trials to drive progress in head and neck cancer immunotherapy

Current curative therapy for head and neck squamous cell carcinoma (HNSCC) involves highly toxic combinations of systemic chemotherapy, surgery and radiation. Unfortunately these treatments are often both “not enough”, with relatively high rates of recurrence following treatment in the setting of locally advanced human papillomavirus (HPV) negative HNSCC; and “too much”, with low rates of recurrence in the setting of HPV positive HNSCC, but often dramatic long term toxicity. Because of the heterogeneity of this disease, incorporation of immunotherapy into the definitive management of HNSCC will require individualized approaches.

Moreover, in addition to HPV, other biomarkers have been linked to outcome in this malignancy, such as TP53 mutation, as well as the expression of multiple individual genes or groups of genes. One gene associated with outcome in HNSCC and of interest to the study of immunotherapy is PD-L1. In one study of several cohorts of HPV negative HNSCC, high PD-L1 expression was correlated with local failure following radiotherapy [21]. Additionally, in this study, patients with low PD-L1/high CD8+ TILs had no local failure or death due to disease. Thus, PD-L1 expression may be a significant biomarker of treatment failure in HPV-negative HNSCC following radiotherapy, which provides a strong rationale for the combination of immune checkpoint blockade and radiation.

However, the question of the optimal integration of anti PD-1 or other immunotherapies into the treatment of HNSCC remains. One attractive means to answer this question is via neoadjuvant or “window of opportunity” trials. This type of clinical trial involves an initial tumor biopsy, short-term treatment with the agent or agents in question, followed by surgical resection. This approach provides vast amounts of data both in regard to potential tumor response as well as immune infiltrate and changes in gene expression. These data may also demonstrate a targetable association between response and immune infiltrate and so may help provide direction with regard to potential predictive markers in HNSCC and choice of treatment.

Using this model, our group has identified several biomarkers of response to immunotherapy. For example, neoadjuvant cetuximab treatment increased the frequency of intratumoral Tregs expressing CTLA-4, which, in turn, suppressed cetuximab-mediated antibody-dependent cellular cytotoxicity (ADCC) and correlated with poor clinical outcome [22]. Additionally, enhanced inflammatory stimulation in the TME using a TLR agonist reduced the induction of Tregs and markers of suppression, including CTLA-4, and increased CD8+ T-cell infiltration into tumors, enhancing the cellular antitumor immune response to cetuximab [23]. Finally, in patients with both HPV-positive and HPV-negative HNSCC, neoadjuvant nivolumab resulted in tumor reductions within one month in nearly half of evaluable patients [24]. These findings point to the effectiveness of a neoadjuvant platform for investigating novel immunotherapy combinations.

### Where are we going with immunotherapy for ovarian cancer?

Despite the successes of immunotherapy with checkpoint blockade in several solid tumors, results from clinical trials in ovarian cancer have been modest, with response rates typically around 10–15% (Table 2). Mechanisms of innate and adaptive immune resistance in ovarian cancer includes the expression of multiple inhibitory receptors, including PD-1, CTLA-4, LAG-3, and TIM-3, on tumors and suppressive antigen-presenting cells. Combined blockade of these pathways may provide superior outcomes compared to PD-1 blockade alone. Dual PD-1 and LAG-3 blockade synergistically enhanced anti-tumor immunity in murine models by increasing the number of functional CD8+ TILs as well as reducing the number of Tregs [25]. Triple PD-1, CTLA-4 and LAG-3 blockade additively increased infiltration of CD8+ T cells and maintained T cell polyfunctionality [26]. However concomitantly targeting multiple immune suppressive mechanisms may result in unacceptable toxicity.

**Table 2.** Reported results of checkpoint blockade in EOC

Agent	Trial number	Disease status	Phase	N	Results (N; duration)
Ipilimumab	NCT01611558	Recurrent	II	40	10% BRR (4; N/A)
BMS-936559 (anti-PD-L1)	NCT00729664	Advanced stage	I	17	6% PR (1; 1.3+ months) 15% SD (3; 6+ months)
Nivolumab	UMIN0005714	Pt-resistant Relapsed	II	2	10% CR (2; 11+ months) 5% PR (1; 11+ months) 30% SD (6; 1 for 11+ months)
Pembrolizumab	NCT02054806	Advanced stage PD-L1^+^	Ib	26	4% CR (1; 6+ months) 8% PR (2; 6+ months) 23% SD (8; 2 for 6+ months)
Avelumab	NCT01772004	Pt-resistant Recurrent	Ib	124	10% PR (12; 4 for 6+ months) 44% SD (55; N/A)
Durvalumab (+ olaparib)	NCT02484404	Recurrent	I/II	10	PR (1; 11+ months) SD (7; 4+ months)

Clinical trials of checkpoint inhibitors in endometrial ovarian cancer

Over 120 clinical trials of PD-1/PDL-1 blockade are ongoing in ovarian cancer, many involving combinations with other checkpoint inhibitors or immunotherapies. Using oncolytic viruses (OV) to reprogram the TME from tolerogenic to immunogenic is a possible strategy but oncolytic viruses have only been partially successful in ovarian cancer. Classes of OV currently being examined in ovarian cancer are Maraba virus armed with tumor antigen, Vaccinia virus armed with CXCR4 inhibitor, and adeno virus with granulocyte–macrophage colony-stimulating factor (GM-CSF).

The CXCR4-CXCL12 axis is pivotal for metastasis and immune suppression within the ovarian TME. CXCR4 overexpression is related to an aggressive phenotype and poor prognosis in ovarian cancer and is essential for cancer-initiating cell (CIC) maintenance, dissemination and metastatic spread to organs where CXCL12 is expressed. CXCL12 stimulates survival and growth of neoplastic cells, promotes tumor angiogenesis by stimulating VEGF and recruiting endothelial progenitor cells to the TME, and attracts Tregs, MDSCs, and peripheral DCs into the TME. However, there is also abundant expression in many cell types, e.g., gastrointestinal and central nervous system.

In murine models, a CXCR4-A-armed OV reduced the tumor load and the immunosuppressive network in the TME, leading to infiltration of CD103+ DCs that were capable of phagocytic clearance of cellular material from virally infected cancer cells [27]. Intraperitoneal delivery resulted in higher accumulation in the TME than systemic treatment. The vaccine-induced antitumor responses inhibited tumor growth and increased OS. OVV-CXCR4-Fc has a multifaceted effect, including direct oncolysis of CICs, decreased recruitment of suppressive elements promoting tumor vascularization, and stimulation of antitumor immunity monitored by the presence of humoral and cellular immune responses to Wilms’ tumor Ag 1 (WT1) expressed by ID8-T cells. OVV-CXCR4-Fc is limited by adaptive resistance that can be overcome by checkpoint blockade and is being investigated in combination with doxorubicin with and without pembrolizumab in a phase I trial.

### Breast cancer immunotherapy: the time has come

Breast cancer can be immunogenic, but most breast tumors are not. Poor prognostic factors (ER-negative, PR-negative, high-grade, lymph node involvement) are associated with higher T cell infiltrates at diagnosis. Higher numbers of CD8+ TILs and a higher CD8+ T cell/FoxP3 + Treg ratio predict better clinical outcomes except in ER-positive breast cancer. ER-positive breast cancers present the challenge of transforming tumors from cold to hot. Triple-negative breast cancer (TNBC) is a good target for cancer immunotherapy, since there are few approved targeted therapies for TNBC, and they are more likely to be infiltrated with immune cells, in particular T cells, and be PD-L1 positive than other breast tumors.

In an open-label phase III study, pembrolizumab monotherapy did not significantly improve OS versus single-agent investigators choice chemotherapy as second- or third-line treatment for metastatic TNBC, although the pembrolizumab treatment effect did increase as PD-L1 enrichment was increased [28]. Atezolizumab has also been investigated in metastatic TNBC in an open-label phase I study in which most patients were heavily pretreated [29]. ORRs were higher in first-line treatment than in subsequent-lines (24 vs 6%). Median OS was longer in patients treated as first-line or with PD-L1 expression of ≥ 1% tumor-infiltrating cells. High PD-L1 expression was independently associated with higher ORR and longer OS.

Atezolizumab was also assessed in combination with nab-paclitaxel chemotherapy versus chemotherapy alone in a phase III study in adavnced TNBC. The addition of atezolizumab to chemotherapy resulted in significant increases in median PFS in the overall population (7.2 versus 5.5 months; HR 0.80, p = 0.002) as well as in patients with PD-L1-positive tumors (7.5 versus 5.0 months, HR 0.62; p < 0.001) [30]. Median OS was also longer in the immunotherapy group than in the placebo group among patients with PD-L1-positive tumors. Among PD-L1-negative patients, the addition of atezolizumab to nab-paclitaxel failed to extend PFS or OS. Adverse events were consistent with the known safety profiles of each single-agent.

PD-L1 is expressed primarily on tumor-infiltrating immune cells in metastatic TNBC and PD-L1 immune cell status predicts PFS benefit for atezolizumab plus nab-paclitaxel. If PD-L1 was expressed on carcinoma cells, the tumors were generally also PD-L1 immune cell-positive [31]. Intratumoral CD8 and stromal TILs were well and moderately correlated with PD-L1 immune cell expression, respectively. CD8+ T cells and stromal TILs predicted clinical benefit only in PD-L1 immune cell-positive patients. The 22C3 and SP142 assays were used to evaluate the tumor specimens, and the SP142 assay identified patients with the smallest HR point estimates and longest median PFS and OS. As such, the SP142 assay at an immune cell 1% cut-off is the only clinically validated and approved test to select patients with metastatic TNBC for atezolizumab plus nab-paclitaxel treatment.

In the neoadjuvant setting, pembrolizumab with standard chemotherapy resulted in a higher pathological complete response (pCR) rate than standard chemotherapy alone [32]. In addition, neoadjuvant pembrolizumab plus chemotherapy followed by adjuvant pembrolizumab showed a favorable trend in event-free survival. In contrast to metastatic TNBC, the clinical activity of pembrolizumab in early TNBC was observed regardless of PD-L1 expression. The underlying immunobiology reflected by this observation is an area of active investigation.

### Implications of immunotherapy treatment in the adjuvant setting

The first adjuvant checkpoint inhibitor immunotherapy trial was with ipilimumab, which showed an absolute difference in survival of 11% versus placebo at both one and five years [33]. This benefit was sustained long-term, with an 8.7% absolute difference at seven years for OS [34]. Pembrolizumab significantly increased one-year rate of recurrence-free survival (RFS) versus placebo (HR for recurrence or death, 0.57; p < 0.001) in the KEYNOTE-054 phase III trial [35]. In the CheckMate-238 trial, the one-year rate of RFS was 70.5% (95% CI: 66.1–74.5) with nivolumab versus 60.8% (95% CI 56.0–65.2) with ipilimumab (HR for disease recurrence or death, 0.65; p < 0.001) [36]. In a cure rate model analysis, the estimated proportion of patients who may never experience relapse was 55% with nivolumab compared to 40% with ipiliumumab [37].

However, the combination of nivolumab plus ipilimumab is more effective than single-agent nivolumab in the metastatic setting, whereas adjuvant immunotherapy approvals for stage III disease are with single agent PD-1 inhibitors. This raises the question of whether the prospect of ‘cure’ for some patients is being compromised by single-agent PD-1 inhibition in the adjuvant setting. There is early evidence of a reasonable response rate to retreatment with PD-1 inhibition following post-adjuvant immunotherapy treatment relapse, with clinical activity in patients with recurrent melanoma treated with anti-PD-1 after adjuvant PD-1 therapy [38]. However, the durability of response is unknown.

Choice of combination anti-PD-1 and anti-CTLA-4 immunotherapy or single-agent immunotherapy is an important consideration i.e. would patients who fail adjuvant PD-1-based immunotherapy have benefited from adjuvant combination PD-1 and CTLA-4 inhibition or single-agent anti-CTLA-4 adjuvant therapy? Moreover, the best treatment for these patients in the metastatic setting is also unclear. In the CheckMate-067 study in patients with metastatic melanoma, patients had improved OS and PFS with nivolumab plus ipilimumab and nivolumab alone versus ipilimumab alone regardless of BRAF mutation status, tumor PD-L1 expression and lactate dehydrogenase (LDH) status [39]. In CheckMate-238, high tumor mutational burden (TMB) and high IFN-γ gene expression signature was associated with improved RFS for both nivolumab and ipilimumab and may represent useful prognostic markers [40]. However, we have no current predictive biomarkers to guide choice of adjuvant immunotherapy.

The impact of toxicity also has to be considered in the adjuvant setting and whether the therapeutic index (i.e. balance of efficacy toxicity) is justifiable in all stage III disease. Adjuvant treatment with ipilimumab is associated with significant toxicity. Nivolumab is associated with fewer toxicities and has similar adverse rates in the adjuvant and metastatic settings. However, while a higher toxicity risk is acceptable in the metastatic setting, in the adjuvant setting many patients may experience significant toxicity who would never have relapsed. These toxicities, although infrequent, can also be chronic and long-term.

Patients failing adjuvant immunotherapy have a reduced chance of survival when metastatic than if they had been treatment-naïve and treated with combination immunotherapy when metastatic. There is also a growing population of patients with long-term toxicity who never needed adjuvant treatment. Improvement in prognostic biomarkers will identify which stage III patients require treatment and will enable adjuvant treatment for stage II disease and appear attainable in the short-term.

### Radiation and immunotherapy in breast cancer

Some of the effects of radiotherapy are now recognized as contributing to systemic antitumor immunity. Recent evidence suggests that radiotherapy can cause immunogenic cell death and facilitate tumor neoantigen presentation and cross-priming of tumor-specific T cells, in effect turning an irradiated tumor into an in-situ vaccine. Thus, radiation can induce responses in tumors that are otherwise immune checkpoint inhibitor-resistant.

The ability of radiotherapy to induce an immune-mediated abscopal effect is likely to depend on its ability to sufficiently alter the TME, so that proimmunogenic effects prevail over immunosuppressive effects. Radiation promotes the priming and effector phases of the antitumor immune response but also activates immunosuppressive transforming growth factor (TGF)-β cytokine and promotes accumulation of Tregs and protumorigenic M2 macrophages (MØ2). Although the positive effects of radiation may predominate, they may be insufficient to shift the balance of the immunosuppressive TME to achieve tumor rejection in the absence of immunotherapy.

TGF-β is a key immunosuppressive cytokine that has multiple effects on cells of the innate and adaptive immune system but, most important for the ability of radiotherapy to prime T cell responses, are its effects on DC and T cells. Antibody-mediated TGFβ blockade during radiation effectively generates CD8+ T cell responses to multiple endogenous tumor antigens in poorly immunogenic mouse carcinomas [41]. In patients with metastatic breast cancer, TGF-β blockade with fresolimumab during radiotherapy was feasible and well tolerated and patients receiving a higher dose had a favorable systemic immune response and longer median OS than the lower dose group [42]. In the mouse model, addition of anti-PD-1 antibodies increased survival achieved with radiation and TGF-β blockade and dual anti-TGF-β and anti-PD-1 blockade may be needed with radiotherapy in breast cancer.

The optimal regimens that should be employed in order to harness the proimmunogenic effects of radiation remain to be defined. It is also unclear whether standard radiation doses and fractionations for a given tumor type should be modified if radiation is to be used to convert the tumor into an *in-situ* vaccine. For example, anti-CTLA-4 caused complete regression of the majority of irradiated tumors and an abscopal effect in mice receiving a hypofractionated regimen (8 Gy × 3) but not in mice treated with a single dose of 20 Gy [43]. In patients with solid tumors treated with pembrolizumab and multisite stereotactic body radiotherapy, partially irradiated tumors showed similar tumor control compared with total tumor irradiation, suggesting an abscopal immune effect of non-targeted radiotherapy and pembrolizumab [44].

Immunotherapy has had modest results in ER-positive breast cancer, but better in the neoadjuvant setting. In the I-SPY 2 trial, patients with HR-positive/HER2-negative breast cancer had an absolute increase in estimated pCR rate of 21% with neoadjuvant pembrolizumab versus standard therapy alone [45]. The safety and feasibility of adding immunotherapy (FLT3L and anti-PD-1) to a combination of radiotherapy and endocrine therapy in the neoadjuvant setting of newly diagnosed HR-positive breast cancer patients is planned.

### The role of immunotherapy in Merkel cell carcinoma

Merkel cell carcinoma (MCC) is a rare, aggressive skin cancer associated with poor outcomes. It is chemo-sensitive, but responses are seldom durable. In 2017, pembrolizumab was added to clinical guidelines after a phase II trial reported that pembrolizumab resulted in an ORR of 56% (4 CRs) in 26 patients with advanced MCC who had received no previous systemic therapy [46]. In an expansion cohort of 50 patients, ORR was 52% in patients with Merkel cell polyomavirus (MCPyV)-positive tumors and 44% in virus-negative tumors [47]. Median PFS was not reached and OS rate at 18 months was 68% (median OS not reached). Nivolumab was also shown to induce durable tumor regressions in the majority of treatment-naive and treatment-experienced patients with advanced MCC, with a manageable safety profile [48].

in the JAVELIN Merkel 200 trial, the anti-PD-L1 agent avelumab was assessed in MCC in patients with ≥ 1 line of chemotherapy or no previous systemic treatment. In previously treated patients, confirmed ORR was 33%, with an estimated 74% of responses lasting ≥ 1 year [49]. One-year PFS rate was 30% and one-year OS rate was 52%. At 3 years, OS rate was 32%, resulting in an OS plateau not seen with chemotherapy and median OS was 12.6 months [50]. There was a trend for higher ORR in patients with a high TMB, with the highest response rates in patients who had tumors that were also PD-L1+ or MCPyV-negative. However, meaningful long-term survival occurred irrespective of tumor PD-L1 expression status. Patients with higher major histocompatibility complex (MHC) class I expression also had a trend for improved response and survival. High response rates and durable responses were also seen with first-line avelumab therapy, with an ORR of 62.1% in a preplanned interim analysis [51]. Among responders, an estimated 93% had a duration of response of ≥ 3 months and 83% a duration of response of ≥ 6 months. In a real-world European expanded access setting, avelumab showed efficacy and safety consistent with these clinical trial results, with a physician-assessed ORR of 54.3% in 105 evaluable patients [52].

Taken together, these data strongly suggest that checkpoint blockade should be the new standard of care for patients with advanced MCC. Clinical trials are now ongoing to assess checkpoint inhibitor therapy in the neoadjuvant and adjuvant settings. Novel combinations are also being assessed, with avelumab in combination with the histone deacetylase (HDAC) inhibitor domatinostat being evaluated in a phase II study in patients with advanced MCC progressing on previous anti-PD-1/PD-L1 therapy.

## Session: drivers of immune responses

### Therapeutic strategies to enhance immune-responsiveness in prostate cancer

The majority of patients with metastatic, castrate-resistant prostate cancer (mCRPC) exhibit primary resistance to immunotherapy. There are multiple mechanisms of resistance to immunotherapy in prostate cancer (PC). These include activation of compensatory feedback immune checkpoint upregulation following immune checkpoint inhibitor therapy, silencing of MHC Class I expression within tumors and release of immunosuppressive chemokines into the TME. However, the DC-based vaccine sipuleucel-T demonstrated a modest survival benefit in prostate cancer, indicating that subsets of patients with mCRPC can benefit from immune-based therapeutic approaches. Furthermore, only approximately 10–25% of mCRPC patients respond to ICB. Therefore, predictive biomarkers to identify responders to immunotherapy in mCRPC are urgently needed. Recent studies have indicated that high TMB, MSI-high) or mismatch repair deficient (MMR-deficient), and CDK12 biallelic loss can predict for responsiveness to immune checkpoint blockade in PC.

Given the resistance to adaptive immune-based approaches to target advanced PC, one strategy to enhance responsiveness to immunotherapy in PC is activation of innate immunity within the TME. Our prior studies have demonstrated that cabozantinib is a tyrosine kinase inhibitor that can activate neutrophil-mediated anti-cancer innate immunity to eradicate aggressive, immunotherapy-refractory PTEN/p53-deficient murine prostate cancer [53]. Cabozantinib resulted in rapid neutrophil infiltration into the tumor bed and induced in vivo tumor cell death via CXCL12-HMGB1-CXCR4-dependent neutrophil recruitment. Given that cabozantinib can trigger a neutrophil-driven innate immune response, its use in combination with treatments that activate adaptive immunity, such as immune checkpoint blockade, may provide durable benefits in mCRPC. Based on this promising data, there are now approx. 20 ongoing and/or planned Phase 2 or Phase 3 clinical trials of cabozantinib with immune checkpoint blockade in mCRPC and other advanced malignancies.

Approx. 25–30% of mCRPCs have defects in the homologous recombination DNA repair pathway [54], thus providing a unique therapeutic vulnerability of this molecular subset of mCRPC to DNA damaging agents. The poly-ADP ribose polymerase (PARP) inhibitor rucaparib has been recently FDA approved for BRCA1 and BRCA2 mutant mCRPC [55]. In addition, the PARP inhibitor olaparib has also been FDA approved in homologous recombination (HRD)-deficient mCRPC [56]. Recent studies have shown that PARP inhibition, which blocks DNA repair, can lead to the accumulation of double-stranded DNA fragments and secondary activation of the DNA-sensing cGAS-STING pathway, resulting in sensitization of homologous recombination repair deficiency (HRD)-deficient breast and ovarian cancers to ICB [57, 58]. These data provide a rationale for using PARP inhibitors as immunomodulatory agents to enhance the efficacy of immune checkpoint blockade in HRD-deficient mCRPC. Clinical trials are ongoing and planned to test PARP inhibitors and STING agonist-based combination therapies to overcome resistance in immune checkpoint blockade-refractory cancers, including mCRPC.

### Checkpoint blockade and immune homeostasis in the gastrointestinal tract

Although effective in patients across many cancers, checkpoint blockade has led to a new class of immune-related adverse events. These immune toxicities can resemble idiopathic autoimmune diseases, such as inflammatory bowel disease (IBD), autoimmune hepatitis, and rheumatoid arthritis, and can provide a window into the biology of immune regulation in humans and potential insight into sporadic autoimmunity.

The gut is the most immunologically complex barrier in the body, host to a diverse commensal microflora and constantly challenged by ingested foreign proteins both of which must be tolerated. The gastrointestinal (GI) mucosa must also defend against pathogenic microorganisms and toxins while maintaining an ability to absorb nutrients. Disruption of immune homeostasis by checkpoint blockade leads to a wide spectrum of common GI toxicities, of which colitis is the most common. This can range from indolent to life-threatening and is the primary cause of treatment-related diarrhea. It is often isolated to the colon, but can involve the GI tract from stomach to rectum [59].

**Fig. 3:**
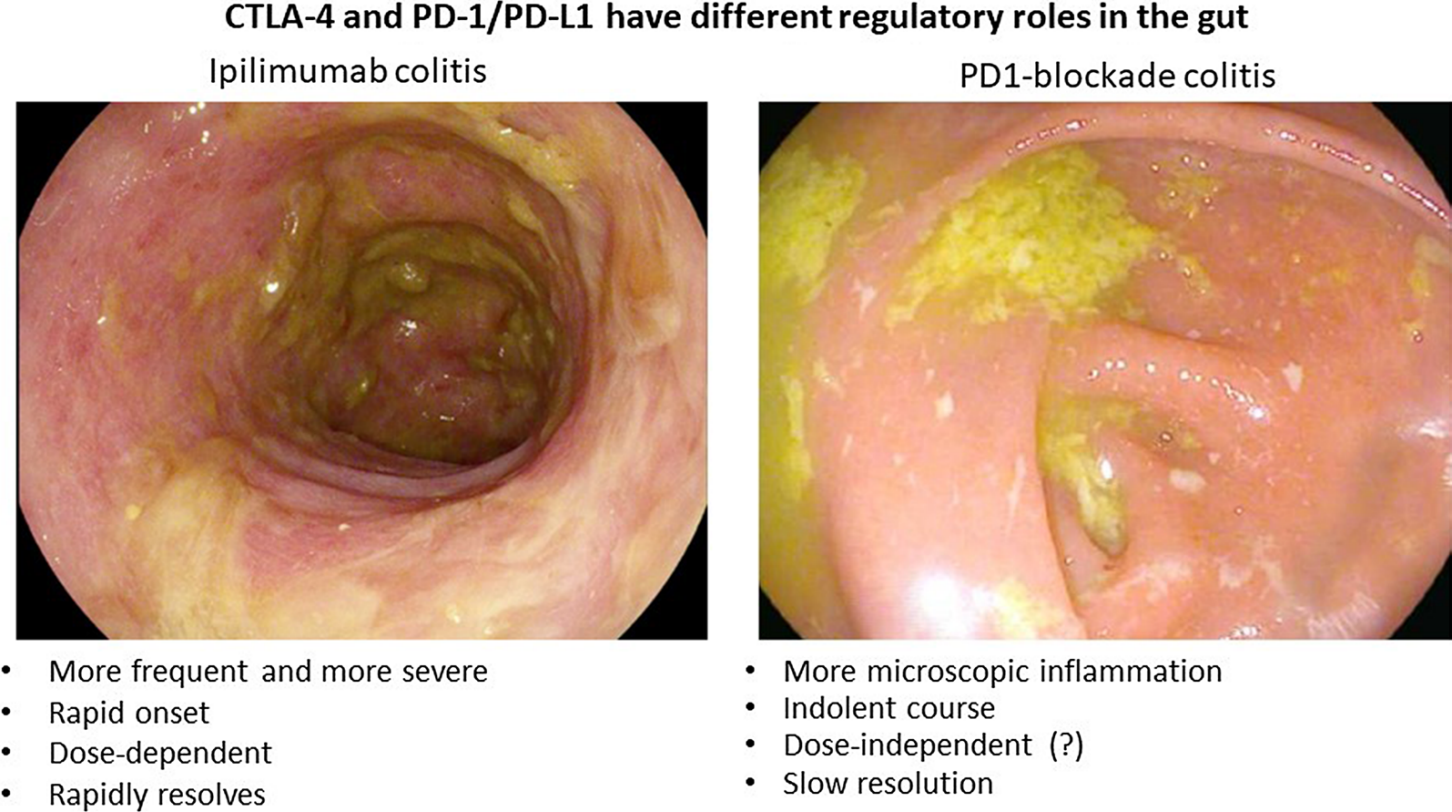
CTLA-4 and PD-1/PD-L1 have different regulatory roles in the gut

CTLA-4 appears to play a more central role in gut homeostasis than PD-1/PD-L1. PD-1/PD-L1 blockade induces colonic inflammation that is clinically distinct from the colitis induced by ipilimumab (Fig. 3) [59]. In PD-1/PD-L1 blockade, colitis is typically less frequent and less severe, with slower onset and resolution. It may also be dose-independent. In many cases, PD-1/PD-L1 blockade colitis is either isolated enteritis or colitis that appears normal on endoscopy and resembles microscopic colitis on biopsy [59]. Ipilimumab-induced colitis most closely resembles pan-colonic ulcerative colitis, a subset of ‘sporadic’ IBD [59].

Prompt diagnosis is the most crucial aspect of management for checkpoint inhibitor colitis. Endoscopic evaluation with biopsy is the gold standard for diagnosis and can provide information on the mucosal severity and extent of disease, which can be used to guide decisions about continuation of immunotherapy. Persistent grade 2 enterocolitis and almost all grade 3–4 enterocolitis will typically require management with systemic glucocorticoids, which are effective in around two-thirds of patients [60] Discontinuation of immunotherapy is also recommended for grade 3–4 enterocolitis. However, there is some evidence that steroids may inhibit the antitumor response [61], so steroid-sparing treatment strategies require further investigation. Glucocorticoids-refractory patients, and patients with recurrent colitis may require the TNF-α inhibitor infliximab, which typically resolves inflammation within 1–3 doses and often with a single dose [60]. TNF-α is a key driver of checkpoint blockade enterocolitis, but does not appear to have an important role in tumor destruction. TNF-α inhibition has been shown to overcome anti-PD-1 resistance in a murine model and infliximab has been associated with a trend toward increased survival in melanoma patients with ipilimumab colitis [62, 63].

In patients with microscopic colitis, local treatment with budesonide may be an appropriate way to avoid systemic steroids. In 38 patients with biopsy-confirmed checkpoint inhibitor enterocolitis, 13 had microscopic colitis and first-line budesonide was effective in controlling microscopic colitis symptoms, allowing most of these patients to avoid systemic glucocorticoids and continue immunotherapy [64].

### Any role for immunotherapy in the treatment of hepatocellular carcinoma?

Patients with advanced hepatocellular carcinoma (HCC) have limited treatment options and poor prognosis. The emergence of immunotherapy, especially checkpoint inhibitors, may afford new therapeutic options. In the CheckMate-259 study, treatment with nivolumab as first-line therapy showed clinically meaningful improvements in OS in patients with advanced HCC, even though the trial did not achieve its primary endpoint of improved survival versus sorafenib (median OS 16.4 versus 14.7 months; HR 0.85, p = 0.0752) [65]. A consistent effect on OS was observed with nivolumab across the majority of predefined subgroups (e.g. hepatitis infection status, presence of vascular invasion and/or extrahepatic spread). Patients with high PD-L1 expression had an increased response rate only in the nivolumab arm, suggesting its potential role as a predictive biomarker. Nivolumab also had a higher ORR (15 versus 7%) with more CRs than sorafenib and a favorable safety profile with fewer toxicity-related treatment discontinuations.

In the KEYNOTE-240 trial of pembrolizumab versus best supportive care as second-line treatment after sorafenib in patients with advanced HCC, both of the co-primary endpoints PFS and OS were improved after a median follow-up of 13.8 months. The risk of death was reduced by 22% in the pembrolizumab arm (HR, 0.78; 95% CI 0.611–0.998; p = 0.0238), although significance was not reached as per the prespecified statistical criteria [66]. ORR was 16.9% with pembrolizumab compared to 2.2% for placebo, consistent with previous data from the KEYNOTE-224 trial, and responses on pembrolizumab were durable (median duration of response of 13.8 months). Post-study subsequent anti-cancer therapy was received by 42% of patients in the pembrolizumab arm and 47% in the placebo arm, which likely confounded the OS data. Safety of pembrolizumab was generally consistent with that previously reported in studies in other cancers.

Despite signs of clinical activity in HCC, response rates with PD-1 inhibitor monotherapy are only around 15–20% so strategies to increase the number of patients who benefit are being explored. VEGF has multiple immunosuppressive effects so the combination of anti-VEGF therapy and checkpoint blockade may increase the antitumor effect. In the ImBRAVE 150 study, first-line treatment with the combination of atezolizumab and bevacizumab significantly improved PFS and OS compared with sorafenib in patients with HCC [67]; risk of death was reduced by 42% (HR 0.58; p < 0.001) and the PFS rate by 41% (HR 0.59; p < 0.001). Median OS was not reached in the combination arm and was 13.2 months with sorafenib alone. Median PFS in patients who received atezolizumab plus bevacizumab was 6.8 months compared with 4.3 months in the sorafenib group. Grade 3–4 adverse events occurred in a similar proportion of patients in both groups and those reported in the combination arm were consistent with toxicities seen with either drug alone.

Another option is pembrolizumab plus lenvatinib, which has been given breakthrough therapy designation by the FDA for patients with advanced unresectable HCC. In a trial of 30 patients, ORR was 37% by investigator assessment using modified Response Evaluation Criteria in Solid Tumors (mRECIST) criteria, and, by independent imaging review, 50% using mRECIST criteria and 37% using RECIST 1.1 criteria [68].

### Driving up immunotherapy response rates in lymphomas

Anti-CD20 antibodies appear to have reached a plateau in efficacy with newer agents offering little in terms of improved survival compared with rituximab. Bispecific antibodies in which two arms link the cancer cell surface to the T cell surface to facilitate killing, such as blinatumomab, mosunetuzemab and REGN1979, offer new promise and may be an option for patients that cannot tolerate, wait for, or afford CAR T cell therapy.

In Hodgkin’s lymphoma, PD-L1 expression on R-S cells corresponds to 9p24.1 amplification. In the first PD-1 trial in lymphoma, nivolumab resulted in an ORR of 87%, including 17% CRs, in 23 patients with heavily treated relapsed or refractory Hodgkin's lymphoma [69]. The rate of PFS at 24 weeks was 86%. In another single-arm study in patients with recurrent classical Hodgkin's lymphoma who had failed to respond to autologous stem cell transplantation and had relapsed after or failed to respond to brentuximab vedotin, independent radiological review committee-assessed objective response was 66.3% and the safety profile was acceptable [70]. However, in patients with relapsed or refractory diffuse large B-cell lymphoma (DLBCL) who were ineligible for or had failed on autologous stem cell transplantation, a low overall response rate was observed [71]. Combination therapy with nivolumab and ipilimumab does not appear to offer any advantage over nivolumab alone in classical Hodgkin’s or B-cell non-Hodgkin’s lymphoma (NHL).

A large number of studies are investigating the use of CAR T therapy in lymphoma. CAR T cell therapies targeting CD19 are a promising approach in NHL and include axicabtagene ciloleucel, tisagenleucel and lisocabtagene maraleucel. Axicabatagene ciloleucel uses CD28 for transmembrane and activation domains, tisagenlecleucel uses CD8 for the transmembrane domain and 4-1BB for costimulation, and lisocabtagene maraleucel uses a CD28 transmembrane domain and 4-1BB for costimulation. All three products are effective in chemotherapy-refractory NHL, with durable responses reported with axicabtagene ciloleucel and tisagenleucel [72–74]. CD28 CAR T cells appear more toxic than 41BB-containing CAR T cells, with neurotoxicity more frequent with axicabtagene ciloleucel, but neurotoxicity is a risk with all three and requires further evaluation.

Despite the clinical efficacy of CAR T cell therapy in relapsed or refractory large B-cell lymphoma, almost 60% of patients relapse or progress because of resistance. Strategies that may further improve the efficacy of CAR T cell therapy include targeting multiple antigens with bi- or multi-specific CAR T cells, combining CAR T cell therapy with agents that overcome immune suppression in the TME (pre- or post-infusion conditioning), and altering the manufacturing process to make the CAR T cells more functional. Various strategies are also underway to improve the safety of CAR T therapy, including the use of synthetic control devices such as inducible CARs, inducible suicide switches such as caspase 9 (iCasp9), or synthetic notch (synNotch) receptors, and IL-blockade using the IL-1 antagonist Anakinra, which can prevent lethal neurotoxicity in vivo.

CAR T therapy may also offer potential in mantle cell lymphoma. In the ZUMA-2 trial, 93% of patients with relapsed or refractory mantle cell lymphoma responded to treatment with KTE-X19 [75]. Responses were durable, with median duration of response not reached at the end of follow-up. Both median PFS and OS were not reached during the study follow-up period; One-year PFS and OS rates were 61 and 83%, respectively. Safety was consistent with that reported with prior studies of anti-CD19 CAR T-cell therapies.

A novel approach in lymphoma is the use of antibody-IFN fusion proteins, which can selectively increase delivery of IFN to the tumor site and reduce systemic toxicity. IGN002, an anti-CD20-IFN-α fusion protein, exhibits enhanced ADCC effector function and superior in vivo anti-tumor activity against B-cell NHL compared with rituximab [76]. A phase I, first-in-human trial is ongoing.

### The host genetic factors as modulators of melanoma immunotherapy outcomes

About 60% of patients do not respond to immune checkpoint blockade and 80% experience autoimmune-like adverse effects that can lead to premature treatment discontinuation. Current biomarkers do not adequately explain immune checkpoint inhibitor resistance or toxicity. Host immune repertoire, including the capacity of host- anti-tumor response has been associated with the signatures of germline genetic variation [77]. In particular, genetic variation in immune-related genes is associated with phenotypes of lymphocyte populations [78] and T-cell differentiation and activity [79]. In fact, > 70% of T-cell specific variation may be explained by cis-acting inherited genetic variation [80]. Thus, the elucidation of baseline capacity of host immunity due to inherited genetic factors, may reveal personalized novel biomarkers and potentially novel actionable targets for improved treatment in melanoma and other cancers. As suggested [81], such germline genetic markers offer the advantages of a rapid and simple blood test, with personalized predictive potential catered to individual patients, due to polymorphic nature of germline single nucleotide variation. Moreover, unlike risk studies of complex traits for common germline variants, the individual associations or polygenic interactions of such germline loci in the context of immune-checkpoint inhibition may exert a large effect size with clinically actionable potential.

A significant evidence strongly supporting a clinical significance of germline factors as novel biomarkers of melanoma therapies and survival has been generated in a large study of immunomodulatory expression quantitative trait loci (eQTLs) in melanoma [82]. It was shown that immunomodulatory eQTLs associate with improved melanoma survival independently of other prognostic and histo-pathological indicators, strongly implicating immune-based germline genetic variation as important and clinical actionable biomarker of prognosis in immunogenic tumors.

Based on this and other supportive evidence, the germline genetic factors have been tested and found to associate with immune checkpoint inhibition response, toxicity and post-treatment survival [81], strongly proposing their capacity to serve as novel biomarkers of immune-checkpoint inhibition. In a recent study, multiple autoimmune risk loci have been found to associate with checkpoint therapy efficacy and toxicity. In 436 patients with metastatic melanoma receiving immune checkpoint inhibitors, the rs17388568 SNP, a risk variant for allergy, colitis and type 1 diabetes, was associated with increased anti-PD-1 response [81]. This variant maps to a locus of established immune-related genes (IL-2 and IL-21), strongly suggesting that autoimmune genetic variation in the pathways involved in cytokine-derived regulation of host immune homeostasis modulates anti-PD1 efficacy.

Driven by these highly promising findings, a collaborative international consortium (IO-GEM) has been established to conduct a first large genome-wide association study (GWAS) of autoimmune toxicity and efficacy related to immune checkpoint inhibition. The goal is to assess approximately 15,000 immune checkpoint-treated metastatic melanoma patients to identify germline biomarkers associated with response, toxicity and survival. Preliminary data on GWAS of anti-CTLA-4 treated patients indicates numerous autoimmune/cytokine and immune responsiveness loci as putative markers predictive of anti-CTLA4 response. For example, IL pathways enriched in GWAS loci are associated with immune-related adverse events, in particular GI toxicity. These data offer a promise for the presence of novel personalized biomarkers of immunotherapy in melanoma or potentially other immunogenic cancers for which the immune checkpoint inhibition emerges as a first-line treatment alternative.

### Targeting cancer through stimulation of innate immunity with intratumoral therapies

Immune checkpoint inhibitors are only effective in subsets of patients with melanoma, lung cancer and other tumor types, while for many common cancers including breast, prostate, ovarian, microsatellite-stable (MSS) colorectal and sarcoma, there is unmet need for novel immunotherapeutic approaches. Data have indicated that tumors can induce type I IFN production by host antigen-presenting cells, required for a T cell response in vivo. Type I IFN signature is also associated with T cell markers in metastatic tumor tissue [83]. This suggests the potential to improve response to cancer immunotherapy by intratumoral activation of innate immunity through the type I IFN response.

Intratumoral therapeutic strategies to target the type I IFN response include agonists of TLR, STING and NLRP3, as well as viral and bacterial-based approaches. The intratumoral OV, talimogene laherparepvec (T-VEC), has been shown to show therapeutic benefit in advanced melanoma [84]. Intratumor oncolytic Newcastle disease virus administration increases local and distant tumor lymphocyte infiltration and expansion of tumor-specific lymphocytes to overcome systemic tumor resistance to immune checkpoint blockade immunotherapy [85]. This suggests that oncolytic virotherapy may improve the efficacy of immunotherapy by altering the TME.

Another approach is the use of bacteriolytic therapy. Spores of *Clostridium novyi* have been shown to germinate in hypoxic tumors and lead to direct tumor destruction. *C. novyi-NT* (non-toxic) is a strain deprived of lethal toxin, which can be used for therapeutic purposes. *C. novyi-NT* induced an immune response against experimental syngeneic tumors which, combined with the bacteriolytic effects of *C. novyi-NT*, eradicated large tumors [86]. Intratumoral injection of *C. novyi-NT* spores was well tolerated in dogs with spontaneous solid tumors, with objective responses in 6 of 16 animals (37.5%) [87]. The most common toxicities were the expected symptoms associated with bacterial infections. On the basis of these results, a human patient with advanced leiomyosarcoma was treated with an intratumoral injection of *C. novyi-NT* spores. There was significant germination with rapid tumor destruction and systemic inflammatory symptoms. In a first-in-human phase I study in 24 patients with injectable treatment-refractory solid tumors, a single intratumoral injection of *C. novyi-NT* spores across six dose cohorts led to germination and resultant tumor lysis of injected tumor masses in 46% of patients [88]. In 22 evaluable patients, 21 (95%) had SD as the best response for the injected lesion (tumor shrinkage of > 10% in 21%) and 19 (86%) had overall SD as the best response per RECIST 1.1. Increased secretion of IFN-γ and TNF-α by circulating T cells was observed, indicating improved systemic tumor specific T cell responses and there was improved immune cell infiltration in metastatic lesions. These signs of improved antitumor activity have led to a trial of *C. novyi-NT* in combination with immune checkpoint inhibitors pembrolizumabs.

### Elucidating metabolic heterogeneity in the tumor microenvironment

It has been suggested that glucose is absent in the TME due to the proliferation demands of transformed tumor cells. However, quantitative mass spectrometry analysis of tumor and paired normal kidney interstitial fluid (IF) demonstrated that this crucial bionutrient is present in RCC TME. The concentration of glucose is comparable (if not greater) between the tumor IF and normal kidney IF. No differences in interstitial glucose was observed between Von Hippel-Lindau wild type and mutant tumors, questioning the notion that hypoxia driven gene signatures drive glucose deprivation in the TME.

Labelling of glycolytic cells with 18F-deoxyglucose (FDG) showed tumor resident cells consume more glucose than the spleen (control) on a per cell basis. This underlies a hallmark of immunometabolism, in which lymphoid and myeloid cell infiltration into tissues is associated with an increase in cellular glycolysis. By magnetic bead separation, we harvested two pure populations from multiple mouse models of cancer: CD45+ immune cell fraction and CD45− tumor cell fraction. Based on these studies, we have found that infiltrating immune cells consuming twice as much FDG as tumor cells on a per cell basis. These hematopoietic cells may be responsible for 55–60% of glucose consumption in the tumor. Enhanced uptake of glucose cells by immune cells was seen across tumor histotypes, genetic backgrounds, and genetically engineered mouse models. Further studies illustrated that the CD11b+ , F4/80+ cells in the MC38 TME consume more FDG than the other tumor resident immune and epithelial cells. Seahorse Mitostress testing confirms tumor-associated macrophages (TAMs) as the most metabolically active cell type in the TME. These mature myeloid cells having the highest basal respiratory capacity at baseline and the greatest spare respiratory capacity when challenged. TAMs also appear to be the have the greatest basal cellular glycolysis glycolytic across biological replicates.

Based on these data, we can conclude that glucose is present in the RCC TME, although whether this is RCC-specific or applicable more broadly to tumors is not yet known. Additionally, metabolite uptake is not equivalent among infiltrating cell types, with TAMs being the most metabolically active cell type.

Clinically, one question left unresolved is whether immunotherapies alter metabolite uptake. FDG PET is not a component of iRESIST criteria for response to checkpoint blockade indicating that clinical PET tools may be tracking both immune responses to cancer and tumor cell growth in the TME. Future work will focus on tracing cellular fate of other nutrients (i.e. glutamine, arginine, or cysteine uptake) in hopes of developing a tumor-specific tracer to appropriately evaluate patient response to IO agents. Preliminary data from our group demonstrated that glutamine uptake is increased in CD45-negative tumor cells. It may be possible to build a model that accounts for different metabolite fate across cell types in the heterotypic TME. Future work will investigate if glycolysis targeting agents inhibit or augment anti-tumor immunity.

### Overcoming anti-PD-1 resistance: a mechanism and a solution

General mechanisms of immunotherapy resistance involve intrinsic tumor biology, including lack of antigen presentation (i.e. lack of antigen expression or lack of antigen processing and presentation); T cell deprived environment (β-catenin, MAPK, etc.); a generalized suppressive microenvironment, and treatment-specific mechanisms (e.g. low PD-L1 expression or JAK2 mutation). Resistance may also be linked to immuno-combination incompatibility or immunotherapy biologic incompatibility.

PD-1 blockade prior to antigen priming of T cell has been shown to abrogate the antitumor immune response and incur primary anti-PD1 resistance, whereas simultaneous anti-PD-1 and proper T cell priming reversed resistance [89]. PD-1 blockade prior to antigen priming with cancer vaccine resulted in induction of dysfunctional T cells and impaired tumor infiltration of antigen-specific CD8+ T-cells.

PD-1 blockade prior to priming with vaccine treatment leads to reduced memory generation and inhibition of downstream T cell signaling. This results in the induction of dysfunctional PD-1 + CD38^high^ CD8+ T cells, which show enhanced apoptosis in the TME. PD-1 blockade of optimally primed CD8 cells prevented the induction of dysfunctional CD8 cells, reversing resistance. Thus, optimal priming of CD8+ T-cells is essential for enhanced anti-PD-1-mediated functionality of cells [89]. In humans, the number of dysfunctional CD8+ T-cells in the tumor and peripheral blood mononuclear cells (PBMCs) correlated with the anti-PD-1 therapeutic response in patients. High numbers of dysfunctional CD8  T cells in the tumor and PBMCs serve as a predictor of failure of anti-PD-1 therapy. Differences in the fraction of PD1 + CD38^high^CD8+ T cells in non-responding versus responding tumor lesions are not due to higher numbers of total CD8+ T cells. PD1 + CD38^high^ CD8 + T cells are a major factor in diminished antitumor activity of activated CD8+ T cells. Anti-CD38 antibody treatment prevents induction of dysfunctional PD1 + CD38^high^CD8+ T-cells in the TME. Treatment with anti-CD38 along with anti-PD-1 results in reversal of anti-PD-1 therapy resistance [89].

Another example of immuno-combination incompatibility is the combination of an anti-check point inhibitor with an agonist antibody. Simultaneous addition of anti-PD-1 to anti-OX40 negated the antitumor effects of both anti-PD1 and OX40 agonist antibody in syngeneic mouse model [90]. Anti-OX40 with a tumor-antigen vaccine led to significant tumor response while the addition of anti-PD-1 negated the effect on both tumor growth inhibition and survival. Antigen-specific CD8+ T-cell infiltration into the tumor was significantly reduced by PD-1 blockade. However, PD-1 blockade did not suppress peripheral antigen-specific immune responses. PD-1 blockade with OX40 costimulation induced apoptosis of CD8+ T cells in vivo. Furthermore, sequential treatment of anti-OX40 followed by anti-PD-1 treatment did not add to the effectiveness of anti-OX40. These results indicate that anti-PD-1 added at the initiation of therapy exhibits a detrimental effect on the positive outcome of anti-OX40 agonist and that sequential therapy may not be adding to the effectiveness of the single agent.

## Conclusions

Immuno-oncology agents have shown potential for clinical application across a broad patient population with both conventional and unconventional response patterns. The success of immune-oncology is strongly correlated with the immune system’s capacity for memory and adaptability, leading to improved survival with the avoidance of selecting resistant forms of tumoral cells. Another advantage is that the efficacy of immunotherapies does not seem to be strongly affected by histology or mutations. However, although immunotherapy has transformed the landscape of cancer treatment, not all patients benefit and either fail to respond or relapse. Extensive research efforts are leading to increased understanding of the complex interactions between the tumor and the host immune response and novel therapeutic strategies across different cancers are being evaluated with the aim of improving patient response rates.

## Data Availability

Not applicable.

## Abbreviations

ACT: Adoptive cell transfer
ADCC: Antibody-dependent cellular cytotoxicity
CAR: Chimeric antigen receptor
CIC: Cancer-initiating cell
CTLA-4: Cytotoxic T-lymphocyte-associated antigen-4
DLBCL: Diffuse large B-cell lymphoma
DCs: Dendritic cells
DNA: Deoxyribonucleic acid
EGFR: Epidermal growth factor receptor
eQTL: Expression quantitative trait loci
ER: Estrogen receptor
FDG: 18F-deoxyglucose
FFPE: Formalin-fixed paraffin-embedded
GI: Gastrointestinal
GITR: Glucocorticoid-induced TNF receptor
GM-CSF: Granulocyte–macrophage colony-stimulating factor
GWAS: Genome-wide association study
HCC: Hepatocellular carcinoma
HDAC: Histone deacetylase
HNSCC: Head and neck squamous cell carcinoma
HPV: Human papillomavirus
HR: Hazard ratio
HRD: Homologous recombination repair deficiency
IBD: Inflammatory bowel disease
IDO: Indoleamine 2,3-dioxygenase
IF: Interstitial fluid
IFN: Interferon
IL: Interleukin
LAG: Lymphocyte-associated gene
LDH: Lactate dehydrogenase
MCC: Merkel cell carcinoma
mCRPC: Metastatic castrate-resistant prostate cancer
MDSCs: Myeloid-derived suppressor cells
MHC: Major histocompatibility complex
MMR: Mismatch repair
mRECIST: Modified response evaluation criteria in solid tumors
MSI-H: Microsatellite instability-high
MSS: Microsatellite-stable
NHL: Non-Hodgkin’s lymphoma
NK: Natural killer
NSCLC: Non-small cell lung cancer
NT: Non-toxic
ORR: Overall response rate
OS: Overall survival
OV: Oncolytic viruses
PARP: Poly-ADP ribose polymerase
PBMC: Peripheral blood mononuclear cell
PC: Prostate cancer
pCR: Pathological complete response
PD-1: Programmed death-1
PD-L1: Programmed death ligand 1
PFS: Progression-free survival
PR: Partial response
RFS: Recurrence-free survival
RNA: Ribonucleic acid
SCLC: Small cell lung cancer
SD: Stable disease
TAM: Tumor-associated macrophage
TCR: T cell receptor
TGF: Transforming growth factor
TIGIT: T cell immunoreceptor with Ig and ITIM domains
TIL: Tumor-infiltrating lymphocyte
TIM: T-cell immunoglobulin and mucin domain
TLR: Toll-like receptor
TMB: Tumor mutational burden
TME: Tumor microenvironment
TNBC: Triple-negative breast cancer
TNF: Tumor necrosis factor
TPS: Tumour proportion score
Tregs: T regulatory cells
T-VEC: Talimogene laherparepvec
WT: Wilms’ tumor
VEGF: Vascular endothelial growth factor
